# Circular bioeconomy in livestock production: harnessing crop by-products in MERCOSUR/MERCOSUL

**DOI:** 10.1093/af/vfaf032

**Published:** 2025-09-19

**Authors:** Griselda Meza Ocampos, Adibe Luiz Abdalla, Arnoldo González Reyna, Tim A McAllister

**Affiliations:** Laboratorio de Biotecnología, Centro Multidisciplinario de Investigaciones Tecnológicas, Universidad Nacional de Asunción (CEMIT -UNA), San Lorenzo, Paraguay; UnilaSalle, Université d’Artois, IDEALISS, ULR, Rouen, France; Centro de Energia Nuclear na Agricultura, Universidade de São Paulo, CCARBON, Piracicaba, Brazil; Biotecnología de la Reproducción en Rumiantes, Universidad Autónoma de Tamaulipas, Tamaulipas, Mexico; Agriculture and Agri-Food Canada, Lethbridge Research and Development Centre, Lethbridge, Alberta, Canada

**Keywords:** circular economy, crops, livestock, best production practices

**Implications**

- Economic significance of Mercosur: As one of the world’s largest economic blocs, Mercosur plays a key role in global food production and trade.
- Regional bioeconomy strategies: Examining Mercosur’s approaches provides insights into successful circular economy practices.
- Policy and future development: Lessons from Mercosur’s experiences can guide policy frameworks to enhance sustainability in livestock production

## Introduction

The increasing global population and the rising demand for sustainable food systems have highlighted the potential of agricultural co-products as viable alternatives for enhancing livestock production efficiency (FAO, 2017). Among agricultural commodities, by-products derived from agro-industrial processing present an economically and environmentally sustainable solution, aligning with the principles of a circular economy (Nath et al., 2023).

**Figure 1.**
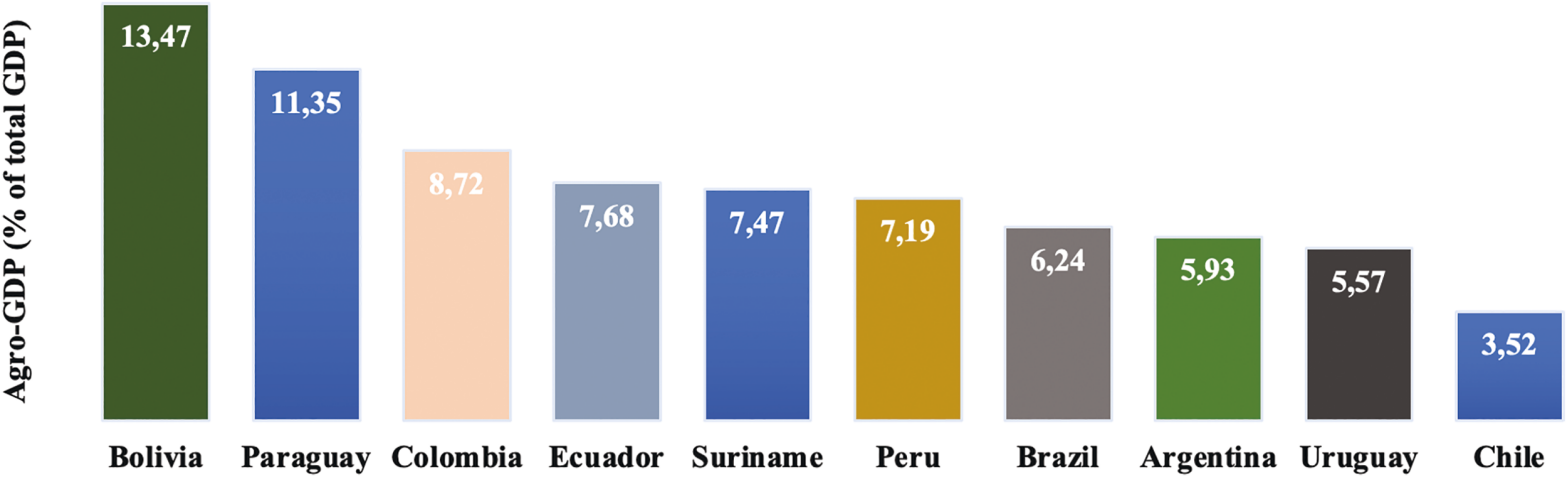
Agricultural value added as a percentage of total national GDP (Agro-GDP) in MERCOSUR countries (2023). Source: World Bank, 2024.

Agriculture is a key economic sector in South America, representing a heterogeneous percentage of Gross Domestic Product (GDP) depending on the country (Pratt and Conroy, 2020; World Bank, 2024; **Figure 1**). Since 1865, the agricultural sector has gained relevance within the South American economy, with the main crops being soybeans (*Glycine max* (L.), corn (maize)—*Zea mays* L.), rice (*Oryza sativa* (L.), wheat *Triticum aestivum* (L.), and sugar *Saccharum officinarum* L.); (Rodríguez et al., 2019).

Southern Common Market or Mercosur is an economic and political bloc that originally comprised Argentina, Brazil, Paraguay, and Uruguay. Mercosur was founded in 1991 to create a common market so as to stimulate development and strengthen democracy, and had early successes, including a ten-fold increase in trade within the bloc in its first decade (Luís and Oliveira, 2012). Venezuela was a member until 2016, but is now suspended indefinitely. Bolivia became a full member in 2024. Chile, Colombia, Ecuador, Guyana, Peru, and Suriname are associate members of Mercosur.

**Figure 2.**
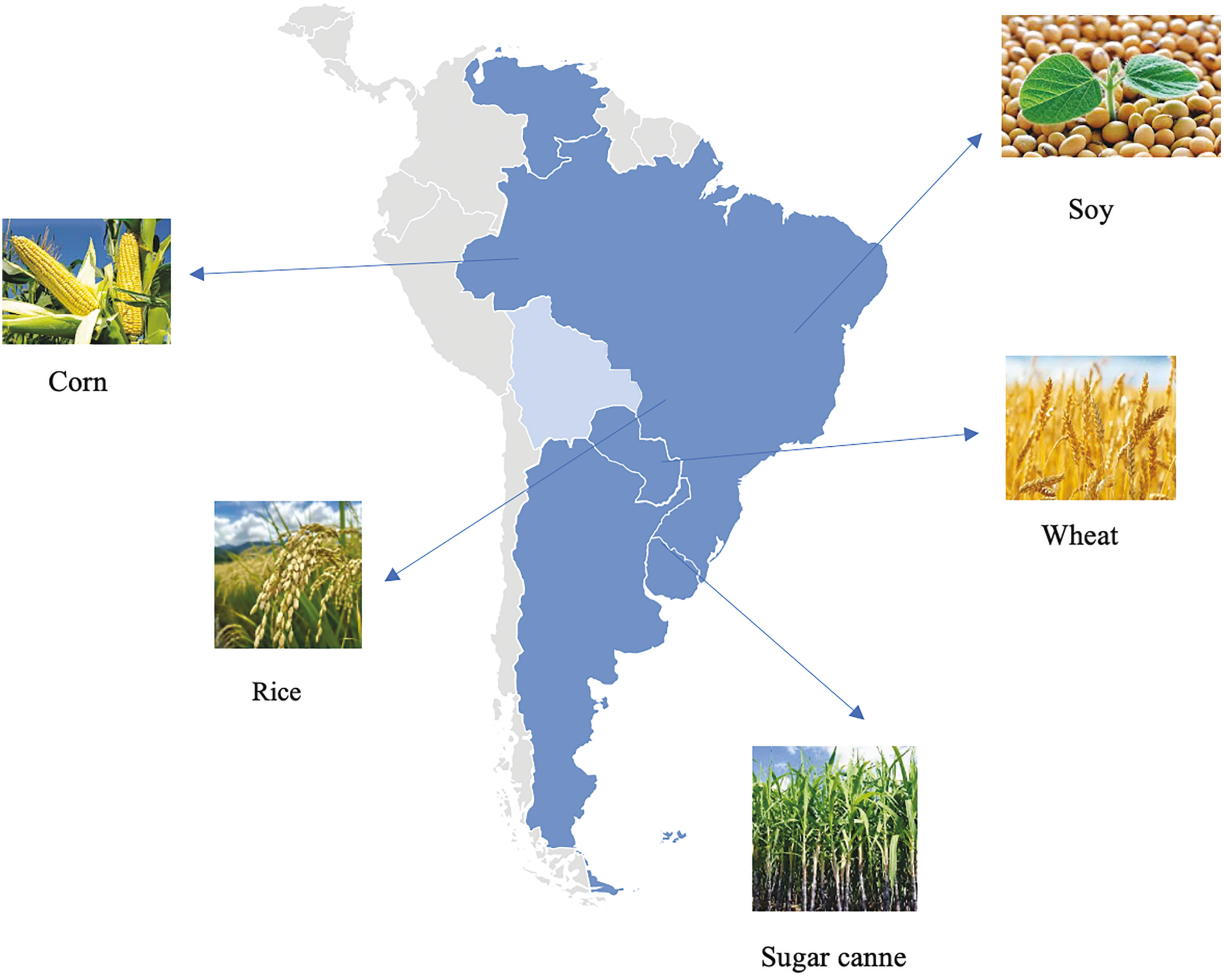
Map of the founding Member States of MERCOSUR and signatories of the Treaty of Asunción are Argentina, Brazil, Paraguay, and Uruguay (blue), and the main agricultural products produced. Source: https://www.mercosur.int/ modified by authors.

For the five major global food crops—soybeans, corn, rice, wheat, and sugarcane. Mercosur is the world’s third-largest producer (Figure 2). Data within the founding members recognized Argentina for its production of soybeans, corn, wheat, beef, and wine, positioning itself as a key player in the global agricultural industry. Brazil stands as the largest global producer and exporter of soybeans, coffee, sugar, and poultry, while also maintaining significant production of beef and corn. Paraguay is one of the leading global producers of soybeans, with soybean meal serving as a key by-product in livestock feed, particularly in poultry and swine production. Uruguay has established itself as one of the world’s leading exporters of rice, barley, rapeseed, malt, soybeans, and beef (USDA, 2024). One of the practical challenges is the use of land for producing crops to feed animals. The use of such crops as feed competes with their use as food and opens the possibility of them being replaced by feed co-products without negatively affecting animal productivity (Herrero et al., 2013; Sandström et al., 2022).

Livestock production is one of the most important economic activities in the member countries of Mercosur (Sterzer and Azizah, 2021). International demand for products derived from livestock has grown, with increasing emphasis on environmental greenhouse gas emissions and animal welfare (Weindl et al., 2017).

The concept of circularity in livestock production systems involves the reuse and recycling of resources within the system, aiming to minimize waste and reduce the environmental footprint of agricultural activities (Velasco-Muñoz et al., 2021). Co-products, which are residues or by-products generated during the processing of food for human consumption, are often rich in essential nutrients and hold significant potential for use in formulating animal diets (FAO, 2024).

Several countries in South America developed bioeconomy strategies, some of which are part of broader regional economic and innovation strategies in response to the European Commission’s call for sustainable production strategies. Others have emerged more from academic, scientific, or national sustainability policy objectives (Garnett et al., 2013; Rodríguez, Rodrigues, and Sotomayor, 2019).

Crop processing by-products, including cereal bran and distillers’ grains (i.e., from biofuels and brewing; Pecka-Kielb et al., 2017), sugar by-products (molasses and beet pulp; Kühnel et al., 2011; Shukla et al., 2022), oilseed meals (rapeseed, soybean, sunflower, palm kernel, sesame, cottonseed, peanut, etc.; Cheng et al., 2022), and citrus pulp (Brambillasca et al., 2013) are viable strategic bioeconomy options.

The objective of this work is to summarize the state-of-the-art regarding the utilization of crop by-products to promote circularity within livestock production systems within the founding members of Mercosur. This analysis aims to identify commonalities and variations in their application across the region. Additionally, the study examines examples of regional bioeconomy strategies, highlighting key approaches to inform future practices and policy development.

### State-of-the-art

Over more than three decades of coordinated collaboration, Mercosur has been established as a major global player in agricultural production and exportation, significantly impacting both regional economies and international markets. The sustained economic growth and development demonstrated by these countries have made Mercosur an increasingly attractive market, which has progressively expanded through the inclusion of new member states (**Table 1**). During the second half of the twentieth century, global soy production grew tenfold from 27 million tons to 269 million tons (WWF, 2016). This increased production was the result of a major expansion in crops, including wheat, corn, sorghum, and canola. Production of these crops by MERCOSUR members is predicted to double by 2050 (Bruinsma, 2009, WWF, 2014).

**Table 1.** *MERCOSUR main* crop *production 2023 -24 (*founding members

MERCOSUR main crop production and forecast 2024/25	Paraguay			Brazil		Argentine		Uruguay	
	Crop	2023–2024 (1,000 Tons)	2024–2025 (1,000 Tons)	2023–2024 (1,000 Tons)	2024–2025 (1,000 Tons)	2023–2024 (1,000 Tons)	2024–2025 (1,000 Tons)	2023–2024 (1,000 Tons)	2024–2025 (1,000 Tons)
Soybean	11.000	11.200	153.000	169.000	48.210	52.000	3.393	3.100
Corn	3.200	5.200	122.000	127.000	50.000	51.000	1.581	1.000
Wheat	893	1.300	8.097	8.100	15.850	17.500	1.344	1.300
Rice	860	900	7.200	8.000	822	950	900	1.050
Rapeseed	105	115	NA	NA	45	35	211	140
Sorghum	108	108	4.426	5.000	2.487	3.600	52	100
Sunflowerseed	39	40	71	90	3.895	4.000	5	10
Cotton	205	250	14.570	16.900	1.650	1.775	NA	NA

#### Paraguay

Paraguay has a total area of 406,752 km^2^ and is divided into two regions by the Río Paraguay: the Oriental Region and the Occidental Region. The economy is based mainly on agriculture and livestock for export. Between 2013 and 2014, approximately 52,381 km^2^ were dedicated to temporary and permanent crops (DGEEC, 2015), particularly soybean (35,000 km^2^), corn (8,000 km^2^), and wheat (5,600 km^2^).

Soybeans are one of Paraguay’s most important crops, and a commercial production of 11 million tons was recorded in 2023–2024 (CAPECO, 2021). The use of soybean by-products plays a crucial role in the production of beef, the country’s main animal export product. Soybean hulls can be included in dairy cattle diets at up to 10% of the total diet dry matter. In January 2025, soybeans and derivatives injected US$338 million into Paraguay’s economy (CAPECO,2025 ; USDA, 2024).

Corn production ranges from 4 to 5 million tons per year. Corn bran, germ meal, gluten, and gluten meal are feed products traded worldwide. Corn bran is commonly used as an energy source for ruminants due to its lower price than grain and its nutritional value tent (the bran is the fiber-rich outer layer that supplies B vitamins, iron, copper, zinc, magnesium, antioxidants, and phytochemicals). Corn bran can be used to completely replace corn grain in concentrate-fed cows without reducing milk quality, although protein supplementation is necessary (Zhang et al., 2021).

Canola (*Brassica napus* (L**.)**, with a production of 60 million tons, has the potential to capitalize on canola byproducts, such as canola meal, for use as animal feed (Yang and Shen, 2018). Sunflower (*Helianthus annuus* (L.) production reached 48 million tons during the 2021–2022 period, reflecting the growing importance of this crop in both oil and by-product production. This production volume contributes to the availability of sunflower meal, which can play a key role in animal feed formulations in this region (Paraguay Sunflowerseed Area, Yield and Production, n.d.).

In 2023, Paraguay exported US$2.8 millionWorth of Cassava (OEC, 2025; OEC, 2025) . Moreover, Cassava (*Manihot esculenta (*Crantz.) processing generates several by-products, such as cassava peels, pulp, and bagasse. The leaves, branches, and peel of cassava can also be used as feed after being dried and ground, following the same process for making cassava flour (Bizzuti et al., 2021).

The use of coconut (*Cocos nucifera* (L.) and almond (*Prunus dulcis* (Mill.) expeller, also known as oilseed cake or meal, as feed for ruminants, poultry, and swine—is a valuable strategy for improving sustainability, reducing feed costs, and utilizing agro-industrial byproducts. Colmán Martínez, (2010) used coconut expeller as a substitute for other feed ingredients in swine diets and demonstrated an improvement in the quality of meat and fat in finished pigs. The digestibility of coconut fed to sheep was evaluated by Corrales et al. (2015). Coconut pulp expeller has also been used as feed for cattle (Gonzalez et al., 2020), and is highly sought after in times of drought, by cattle ranchers in the Chaco (McDonald, 2007) . Almond expeller is used as feed for pigs (Ahammad et al., 2024; De Ciencias et al., 2015), chickens (Moradi Yeganeh et al., 2020), and tilapia (Amarowicz et al., 2005).

#### Brazil

Brazil has an approximate area of 8,515,767 km² and is divided into five major geographic regions: North, Northeast, Center-West, Southeast, and South. The North region includes much of the Amazon rainforest, while the Center-West features areas like the Pantanal and the Cerrado. The Southeast and South regions are the most industrialized and agriculturally developed. Agriculture and livestock are cornerstone sectors in Brazil’s economy, contributing significantly to its GDP and reinforcing the nation’s status as a global leader in agribusiness. The country also excels in crop and plant co-product production, dominating global markets for soybeans, sugarcane, coffee, and orange juice.

Soybean production alone supplies 14% of the global market, highlighting Brazil’s role in enhancing global food security (CONAB, 2023) . Advancements in technology and sustainable practices have been instrumental in maintaining high productivity while addressing environmental challenges. Precision agriculture and integrated crop-livestock-forestry systems (ILPF) have improved resource efficiency and reduced greenhouse gas emissions, enhancing the sector’s economic sustainability (EMBRAPA, 2024) .

The growing demand for sustainable agricultural systems has heightened interest in the efficient utilization of by-products from Brazilian agriculture as animal feed. These co-products, derived from crop processing and agro-industrial activities, present significant opportunities to minimize waste, enhance resource efficiency, and promote bio-circularity within livestock systems. Among the most commonly utilized co-products are soybean meal (41 million tons/year), cottonseed meal (6.8 million tons/year), pelleted citrus pulp (16 million tons/year), and soybean hulls (USDA, 2022). These by-products serve as essential sources of protein, energy, and fiber, thereby making substantial contributions to sustainable livestock production practices

The incorporation of co-products as feed ingredients exemplifies bio-circularity by promoting waste valorization and reducing environmental footprints. For example, Cassava by-products, such as cassava peels and cassava bagasse, have been identified as valuable feed sources for ruminants. Bizzuti et al. (2021) reported that cassava by-products serve as energy-rich alternative feeds due to their high starch content. The study also highlighted their accessibility and cost-effectiveness, particularly in regions with substantial cassava production. These findings align with the principles of bio-circularity, as they valorize agricultural residues and reduce waste.

By-products from the biodiesel industry, including glycerin and oilseed meals, are increasingly being incorporated into ruminant diets. Oliveira et al. (2012) highlighted the nutritional advantages of these by-products, particularly their high energy and protein content. Additionally, the study emphasized their role in reducing feed costs and improving resource efficiency, thus contributing to a circular economy within livestock production systems.

Cottonseed meal and cake are well-recognized protein sources in livestock diets. Research by Paim et al. (2016a, 2016b) investigated the inclusion of cottonseed co-products in the diets of rams during the peripubertal period. These studies demonstrated the safe use of cottonseed by-products without adverse effects on reproductive health, highlighting their potential to enhance feed efficiency while reducing reliance on conventional feed resources.

Various agricultural by-products, such as fruit and grain residues, have also been evaluated for their effects on rumen fermentation and methane production. Bizzuti et al. (2023) investigated the degradability and methane emission reduction potential of several by-products, demonstrating their ability to reduce methane without compromising nutrient availability for ruminants. These findings underscore the environmental benefits of incorporating co-products into animal diets, which is a critical aspect of bio-circular agricultural practices.

Lipid supplementation has emerged as a promising strategy for mitigating enteric methane emissions in ruminants. Takahashi et al. (2024) investigated the use of macadamia (*Macadamia integrifolia)* by-products as a lipid source in sheep diets, reporting significant reductions in methane emissions. Complementing these findings, Marconato et al. (2021) evaluated the use of increasing levels of macadamia nut cake in lamb diets. Their study demonstrated that macadamia nut cake can be incorporated without compromising growth performance, carcass traits, or meat quality, underscoring its potential as a sustainable feed alternative. This innovative strategy highlights the potential of lipid-rich agricultural residues to mitigate environmental impacts while maintaining livestock productivity (Marconato et al., 2021; Takahashi et al., 2024)

Brazil’s agricultural co-products represent valuable resources for advancing sustainable livestock production systems. From cassava by-products to biodiesel industry residues, these materials support cost-effective and environmentally friendly feeding practices. Furthermore, their integration into livestock systems aligns with the concept of bio-circularity by transforming agricultural residues into high-value inputs, thereby closing the loop within agricultural ecosystems. Continued research and policy support are essential to expand the adoption of these practices, ensuring their scalability and long-term sustainability in Brazilian agriculture.

#### Argentina

Argentina covers about 2,780,400 km² and is divided into six major regions: Northwest, Northeast, Cuyo, Center, Patagonia, and Pampas. The Pampas region is the main agricultural and livestock area of the country.

Argentina has been one of the major grain exporters since the early 20th century (Aramburu Merlos et al., 2015). Assuming a standard nutritional unit of 500 kg grain equivalent per capita per year, it produces enough grain to feed ca. 200 million people (Connor et al., 2006). Argentina currently contributes 20% of global soybean production, and 2% of both corn and wheat global production, and it is among the main exporters of grain and milling products from these crops in the world market (Andrade and Satorre, 2015).

Since the early 90s, overall grain production has nearly tripled. Wheat, corn, and soybean alone account for more than 90% of production, with over 20 million hectares cropped to soybeans every year. Argentina is third in soybean exports and the leading exporter of soybean cake, oil, and biodiesel. It is the second and sixth exporter of corn and wheat, accounting for 78% of total crop area (FAOSTAT and FAO, 2015). Since its internal food demand is expected to remain unchanged, any future increase in crop production in Argentina will result in a parallel increase in exports (Alexandratos and Bruinsma, 2012).

There is an estimated 30.000 ha of cassava grown in Argentina. Cassava is grown for its roots for human food (Latif and Müller, 2015), whereas the aerial parts (leaves and stems) are generally fed to livestock (fresh, dried, or as silage; Leguizamón et al., 2021; Mukhtar et al., 2023)

Argentina holds the third position in global peanut exports, with more than 70% of Argentina’s peanuts being exported because of their recognized high quality (USDA, 2024). Production of nuts (mainly almonds, pistachios (*Pistacia vera* (L.), hazelnuts (*Corylus avellana* (L.) and walnuts (*Juglans regia* (L.) for human consumption produces a variety of by-products, including: shells, skins (perisperms), and oil cakes. Nut by-products generally have a low moisture content, which makes them easy to handle and store (Alibés et al., 1983). Nut-derived by-products contain a range of bioactive constituents, including phenolic compounds, polyunsaturated fatty acids, and essential vitamins, which have the potential to promote animal health and improve the nutritional and functional properties of animal products (Aguilar et al., 1984; Alkhtib et al., 2017; Caccamo et al., 2019; Musati et al., 2023).

Cotton (*Gossypium hirsutum* (L.) cultivation generates a high quantity of by-products potentially usable as ruminant feed. Cotton plant by-products consist of leaves, bracts, carpels, stems, linter, and seeds (Arroquy et al., 2018). Cotton gin trash, cottonseed hulls, and cotton textile mill waste also have potential as feed, especially for ruminant livestock located near cotton and cottonseed processing facilities (Wang et al., 2023).

#### Uruguay

Uruguay has a total area of approximately 176,215 km² and is divided into 19 departments, without larger official geographic divisions. The Pampas plain region predominates, featuring a temperate climate ideal for agriculture and livestock.

Uruguay boasts 16.4 million hectares for agricultural use, covering more than 90% of its territory (DIEA, 2024) . The total cultivated area (including winter and summer crops) in the 2022–2023 season topped 2.25 million hectares, which represented a 14% increase compared to 2021–2022 (Agriculture Report, 2024). Green manures (cover crops), crop rotations, compost, manure, and biofertilizers are widely used practices within the Agroecology Network of Uruguay (RAU, Red de Agroecología del Uruguay) farms (Khan et al., 2024).

Uruguay enjoys a significant reputation as a sustainable producer of food and fiber for the rest of the world. For decades, crop production has been carried out in rotation with pastures and other cash crops. The agro-industrial sector plays a crucial role in Uruguay’s export matrix. With a population of 3.5 million, the country exports enough products to supply nearly 30 million people. Uruguay’s international prestige as a reliable supplier of food and agricultural products allows it to access 160 markets, consolidating its position as one of the main global exporters of rice, barley, rapeseed, malt, and soybeans (Baeza et al., 2022; Pereyra-Goday et al., 2022).

Soybeans are one of the three main export products of Uruguay, which positioned itself as the fifth exporter of soybeans in the world in 2022, with 3 million tons. In 2023, the total exported soybeans were 773 thousand tons, worth around US$411 million. Uruguayan wheat production in 2024–2025 is forecasted at 1.3 million tons, 260,000 tons lower than the previous crop season, which saw record high yields . Soy flour and expeller from oilseed processing are also by-products that are used in livestock feed. From soybeans, there are also flour and soybean cake, which vary in nutritional value depending on whether the oil is extracted using solvents or just by pressing (Barley Manufacturers in Uruguay, Barley Wholesale Suppliers and Exporters in Uruguay, n.d.; Tseng et al., 2021).

Rice is the major food crop, followed by wheat and sugarcane. Corn is the principal feed concentrate. Barley, oats, and sorghums, along with oil crops (flaxseed and sunflower seed) and sugar beets, are also important crops (Tseng et al., 2021).

Corn accounted for 15% of the summer crop area in 2022–2023, with a total of 188,000 hectares cultivated. Wheat accounted for 35% of the winter crop area during the 2022–2023 harvest. Wheat is the third most valuable crop exported in recent years. Its exports had strong growth in 2022 and 2023 and totaled US$244 and US$234 million, respectively (Bustamante-Silveira et al., 2021; Paruelo et al., 2024). Wheat bran, a by-product of wheat flour, is also a frequent component of balanced rations.

Barley is mainly destined for the production of malt for later export. Brewing barley is planted under contract with malting plants, which export the product mainly to Brazil. About 5% of barley is used in the local market for the production of seed. To produce beer, barley grain must first be malted, with Uruguay ranked as the sixth largest exporter of malt in the world in 2023.

In Uruguay, sunflower is primarily cultivated for seed oil production, covering approximately 150.000 hectares. However, its potential for integration into animal production systems as an alternative whole-crop silage has also been recognized (Cozzolino et al., 2002).

## Bioeconomy as an Innovation in MERCOSUR/MERCOSUL

**Figure 3.**
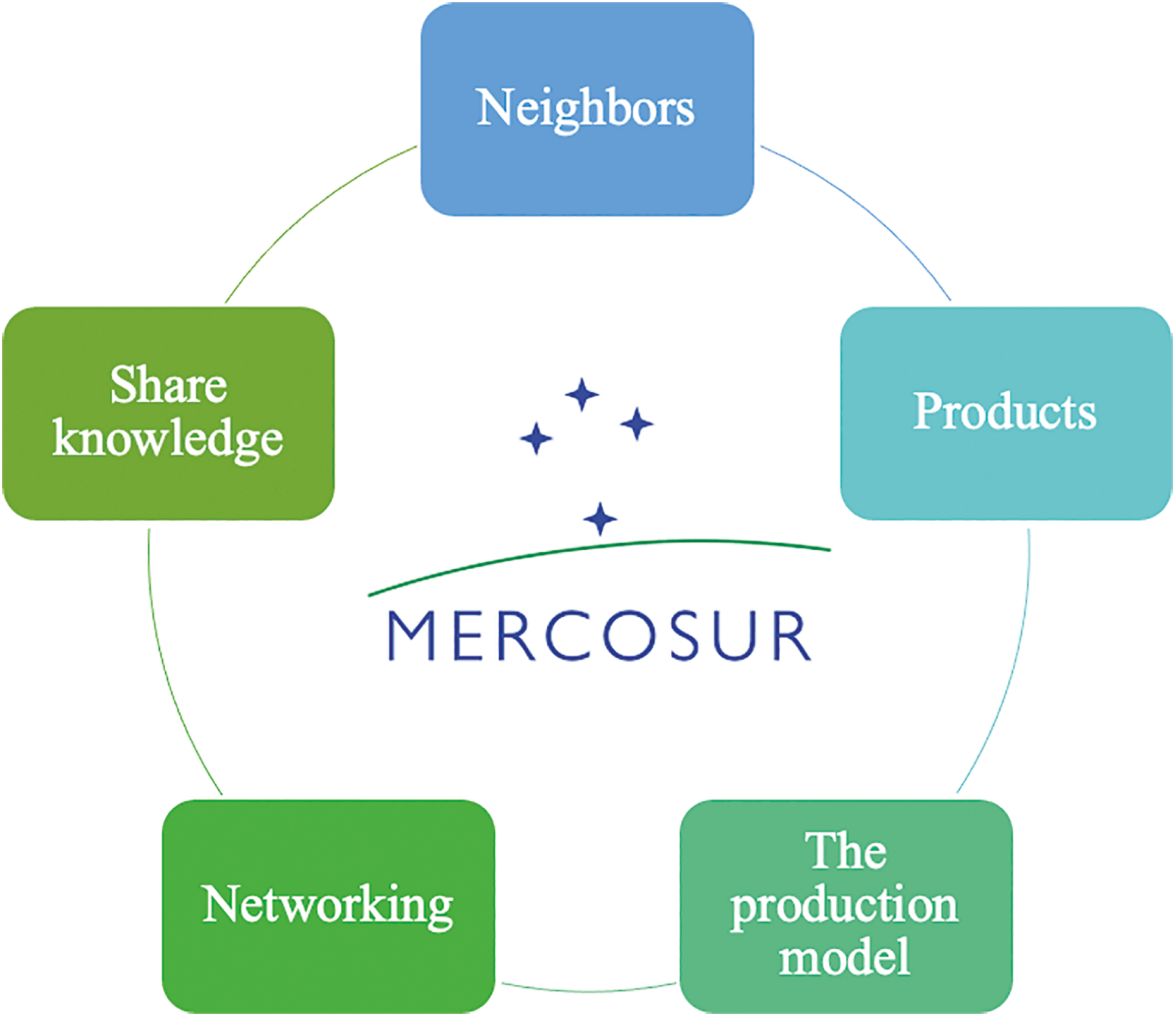
Mercosur bioeconomy-based strategy.

The concept of bioeconomy is centered on the efficient valorization of renewable resources and presents a unique opportunity for Mercosur countries owing to their endowment of natural resources (biomass), technological abilities, industrial facilities, and complementary assets in various productive networks (i.e., food, bioenergy, etc.). These are all factors that can contribute to the construction of a competitive economy. This strategy is particularly relevant to this region for the following reasons: a) *Neighbors,* the advantage of location; b) *Product*, biomasses are generated in ecosystems; c) *The production model* is based on production/reproduction of biomass and transformation of the main products; d) *Networking*, the organizational and operational supports; and e) *Share knowledge*, such as production networks share critical assets—technologies, processes and operating routines (Bisang and Regunaga, 2022; **Figure 3).**

## Strengths of Member Countries to Implement Bioeconomy Strategies and Improve Processes for Animal Nutrition

To fully capitalize on the opportunities offered by the bioeconomy, simply having abundant biomass resources is not enough. It is essential to create a robust framework that includes well-trained human resources and a solid research infrastructure, particularly in biotechnology and process innovation. This is further supported by the land use and management policies in Paraguay, Brazil, Argentina, and Uruguay. While each country adopts a unique approach influenced by its specific geographical and environmental conditions, they share common goals, such as promoting sustainable agriculture, protecting forests, and managing ecosystems effectively (**Table 2**).

**Table 2.** Land use and management policies

Land use and management	Paraguay	Brazil	Argentina	Uruguay
Objective	Promote sustainable agriculture and forest protection in the Chaco and Eastern region	Protect Amazonia and promote sustainable agriculture in intensive agricultural	Reduce deforestation in the Gran Chaco and optimize agricultural land use	Sustainable agriculture with focus on responsible livestock production and soil protection
Conservation areas	Tropical forests of the Chaco and Eastern region	Amazonia, Pantanal, Cerrado	Gran Chaco forests and wetland areas	Protected areas and natural ecosystems along the coast
Conservation approach	Protect ecosystems and biodiversity	Protect tropical forests and water resources	Control deforestation and manage forests sustainably	Protect rural ecosystems and reduce soil erosion
Regulatory instruments	National Forest Law and land-use regulations	Brazilian Forest Code, Environmental Licensing System	Native Forest Law, reforestation programs	Land Use Planning Law and Sustainable Land Use Law

**Figure 4.**
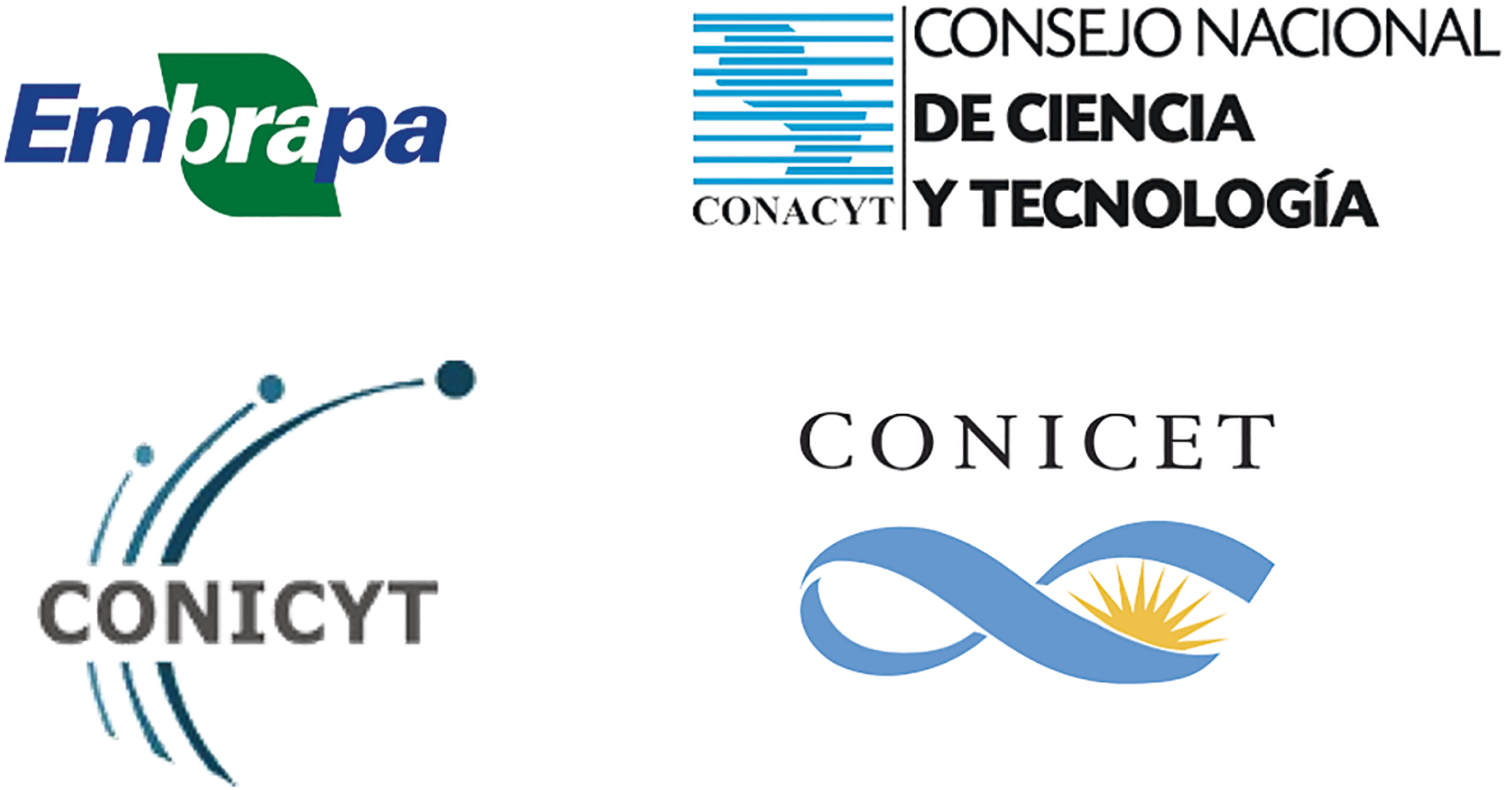
Research institutions from Argentina, Brazil, Paraguay, and Uruguay.

In the Mercosur countries, various public institutions dedicated to science and technology implement specific development and innovation programs (**Figure 4):** Brazil, Brazilian Agricultural Research Corporation (EMBRAPA). Paraguay, the National Council of Science and Technology (CONACYT), Argentina has the National Council for Scientific and Technical Research (CONICET), and Uruguay National Council for Innovation, Science and Technology (CONICYT).

## Benefits of Using Crop By-products in Livestock Systems

**Figure 5.**
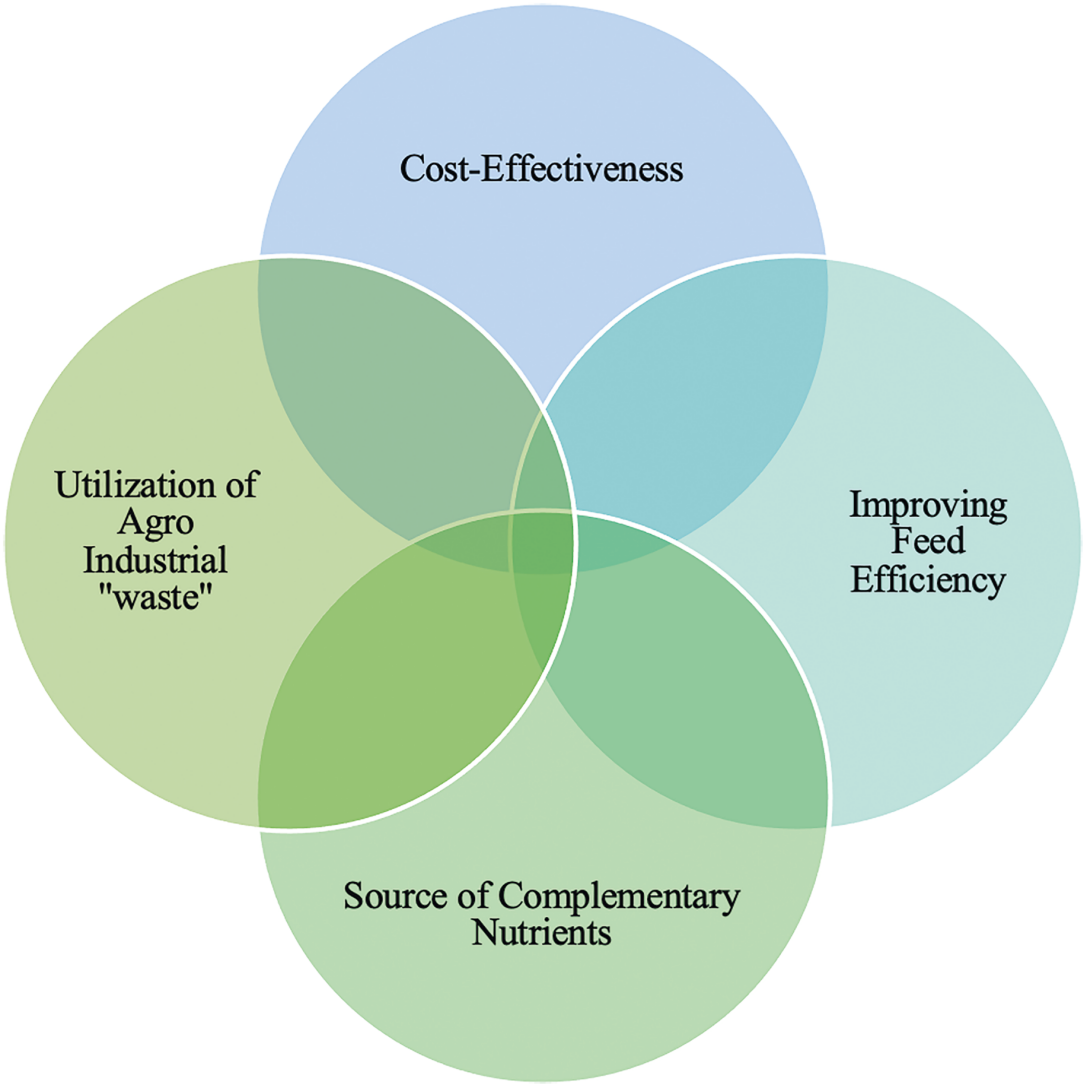
Benefits of using crop by-products in livestock systems.

By utilizing crop by-products, farmers can lower feed costs, as these by-products are often less expensive than conventional feed ingredients. The use of crop by-products helps to reduce waste and supports sustainable agricultural practices. It also minimizes the environmental footprint of livestock farming by making use of what would otherwise be discarded. Crop by-products are not always nutritionally complete on their own but can be used effectively as part of a balanced feed formulation, complementing other high-protein and energy-rich feeds. Some crop by-products, such as rice bran, corn stover, and peanut hulls, provide important fiber that supports ruminant digestion, while others, like molasses, offer quick and readily available energy. By processing crop by-products into high-value livestock feed ingredients, farmers and feed manufacturers can create new products and markets, contributing to the development of the agricultural sector (**Figure 5**).

## Conclusion

The integration of crop by-products into livestock production systems in MERCOSUR is a fundamental step toward achieving greater circularity in agriculture. By reusing these materials, farmers can reduce feed costs, minimize waste, and enhance the sustainability of their operations. This not only supports the economic viability of livestock farming but also contributes to environmental stewardship, positioning this region as a key player in the global move toward more sustainable, circular agricultural systems. Although comprehensive, up-to-date data on this specific integration can be challenging to obtain due to variations in country-specific practices, economic conditions, and agricultural policies, there are several key trends, statistics, and observations that provide insight into the use of crop by-products in livestock systems across the region.

## About the Authors

**Dr. Griselda Asunción Meza Ocampos** is an Associate Professor in Veterinary Medicine at UniLaSalle, Rouen, and the Faculty of Agricultural Sciences (FCA-UNA), where she teaches undergraduate and postgraduate courses. She is also an investigator at the Multidisciplinary Center for Technological Research (CEMIT) at the National University of Asunción (UNA), Paraguay. She holds a Doctor of Veterinary Medicine (DVM) from the National University of Asunción (UNA), Paraguay, and a Master’s in Epidemiological Surveillance in Humans and Animals (UPSaclay). She completed her PhD in Toxicology, Genetics, and Nutrition at the National Polytechnic Institute of Toulouse (INP), France. Her research interests include sustainable livestock production, alternatives to chemical parasite control, and the use of medicinal plants for veterinary applications. She is particularly focused on evaluating plant-based anthelmintic properties and strategies to reduce the environmental footprint of animal production.

**Dr. Adibe Luiz Abdalla** is an Associate Professor at the University of São Paulo, based at CENA/USP. He holds a degree in Agronomy and specializes in Animal Nutrition and the application of isotopes in ruminant metabolism. He completed postdoctoral research at Reading University (UK) and UC-Davis (USA). His research focuses on sustainable ruminant production, including the mitigation of greenhouse gas emissions, alternative diets, and isotopic models for metabolic and sustainability assessments. He has contributed to the advancement of phytogenic additives, silvopastoral systems, and alternative feed supplements to enhance production efficiency. At CENA/USP, he integrates isotopes techniques, rumen microbiology, and sustainable management into his teaching and research. His work aligns with global efforts to promote climate resilience in livestock systems.

**Dr. Arnoldo González-Reyna** is an Emeritus Professor in Applied Reproductive Technologies (ART´s), at the Autonomous University of Tamaulipas (UAT), Mexico. He is an expert in animal reproductive physiology and reproductive biotechnology. He has extensively worked on improving livestock production systems, focusing on ruminant reproduction and optimizing biotechnology applications in animal husbandry. González-Reyna obtained his PhD in Biology with a specialization in Endocrinology and Physiology of Reproduction in Ruminants from the University of Saskatchewan, Canada. His research has had a significant impact on reproductive biotechnology and genetic improvement in livestock species, especially ruminants as hair sheep and goats. In addition to his educational work, Dr. González-Reyna has collaborated with various international and national institutions, promoting the development of innovative strategies for sustainable animal production.

**Dr. Tim McAllister** received an M. Sc. in Animal Biochemistry from the University of Alberta in 1987 and a Ph.D. in Microbiology and Nutrition from the University of Guelph in 1991. He has worked as a research scientist with Agriculture and Agri-Food Canada since 1996, where he now holds the position of principal research scientist in microbiology and beef cattle production. His team has addressed a variety of topic areas related to optimizing the role of livestock in a circular bioeconomy. These include assessing and mitigating greenhouse gas emissions from agricultural systems, optimizing manure management, utilizing food loss and waste as feed, promoting food safety, and recognizing the role of grassland ecosystems in sustaining biodiversity. His team has been the recipient of numerous societal awards for their contribution to beef cattle production in North America.
